# Supplementary material for: Here Puppy, Chew on This: Short-Term Provision of Toys Does Not Improve Welfare in Companion Dogs
Source: Animals (Basel). 2023 Oct 27;13(21):3340. doi: 10.3390/ani13213340 (PMC10648485; doi:10.3390/ani13213340)
Supplement: Supplementary file 1 [file animals-13-03340-s001.zip › Supplementary Materials File S1.pdf]

Supplementary Materials for Here puppy, chew on this: Short-term provision of toys does not improve welfare in companion dogs

Kelly Chan, Carol Arellano, Alexandra Horowitz

File S1: Supplemental methods for cognitive bias test procedures and analyses

Testing procedures

An experimenter met subjects and their people at a street-level entrance of the College and accompanied them to the testing space in the Dog Cognition Lab. Upon arrival, the dog was allowed to explore the room independently, off-leash, for up to five minutes while the experimenters explained the study to the subject's person. This allowed subjects to acclimate to the novel environment prior to starting the trials.

The stimulus array consisted of three locations; A center location 0.5 m away from the experimenter and a left and right location, each 0.5 m away from the center location. In the P- and N- trials, a bowl was placed at left or right locations in the stimulus array and were baited or unbaited, depending on the trial type. Large pieces of of PureBites beef liver treats were broken up into several smaller pieces and one of the smaller pieces was used to bait the bowl for the P-trials. In the A-trials, the bowl was always unbaited and placed in the center location of the stimulus array.

For all training and testing trials, the experimenter baited or pretended to bait the bowl. In order to match sensory and bodily cues directed at the subjects for all trial types, the experimenter followed the same hand gestures (e.g. opening of the bag of treats, briefly placing her hand in the bowl, etc.) to prepare the bowl. She gathered the subject's attention forward by using dog-directed speech and said, "Hi, puppy!" three times before stepping 0.5 m towards the stimulus array, placing the bowl down in the location appropriate to the trial, and returning to her central position 0.5 m away from the stimulus with her gaze downward. Once back to her starting position, the experimenter would give a verbal cue, "ok!", for the humans to drop their dog's leash or remove their hand from their body or collar. Humans were instructed to refrain from gesturing or verbally or physically prompting investigation upon releasing their dog. Similarly, experimenters were instructed to refrain from gazing at the stimulus or the subject and interacting with the subject during the trials. These procedures were intended to mitigate human-induced bias on subjects' in-test behaviors.

Subject were allowed 30 s to freely approach for all trials. If they did not visit the bowl within thirty seconds, they moved to the next trial. If they did not approach the bowl over three consecutive trials, their participation in the study was concluded.

Post-hoc behavioral analyses

Adjusted averaged latencies:

In addition to calculating the averaged latencies, we performed an adjustment that takes into consideration inherent differences in subjects' speed to approach in the different stimuli (determined by their time to approach in the P and N trials). Raw latency scores were adjusted using a formula (see below) derived from Mendl et al. [1]. In the event that the subject did not approach the P or N trial during the testing phase, the last P or N trial from the training phase was used in the adjustment.

Adjusted A-latency = $$\frac{(Latency\ A - Latency\ P)}{(Latency\ N - Latency\ P)} * 100$$

First A-trial:

Subjects were presented with six trials to the ambiguous stimuli. To account for potential learning effects, in which subjects learned over the six trials that the ambiguous bowl contained no food, we also analyzed subjects' latency to approach in their *first* A-trial and compared differences in results between subjects' average and first A-trial latencies. Latency data in these analyses were not adjusted.

Supplemental References:

S1. Mendl, M.; Brooks, J.; Basse, C.; Burman, O.; Paul, E.; Blackwell, E.; Casey, R. Dogs Showing Separation-Related Behaviour Exhibit a "Pessimistic" Cognitive Bias. *Current Biology* **2010**, *20* (19), R839–R840. [CrossRef]
