# Supplementary material for: Here Puppy, Chew on This: Short-Term Provision of Toys Does Not Improve Welfare in Companion Dogs
Source: Animals (Basel). 2023 Oct 27;13(21):3340. doi: 10.3390/ani13213340 (PMC10648485; doi:10.3390/ani13213340)
Supplement: Supplementary file 1 [file animals-13-03340-s001.zip › Supplementary Materials Table S1.pdf]

Supplementary Materials for Here puppy, chew on this: Short-term provision of toys does not improve welfare in companion dogs

Kelly Chan, Carol Arellano, Alexandra Horowitz

Table S1. Subject demographic data

Dog name	Age (yrs)	Sex	Breed	Reported Favorite Toy	Group, Level from 1-3 (if applicable)
Arlo	5.33	M	Mixed	Plush toy	E, 2
Bella	2.42	F	Mixed	Ball	C
Beto	1.92	M	Mixed	Food stuffed toy	C
Cash	4	M	Mixed	Squeaky toy	E, 1
Chaco	4.33	M	Australian Cattle	Ball	E, 1
Clover	0.5	F	Mixed	Plush toy	E, 2
Cody	1.92	M	Mixed	Food stuffed toy	E, 3
Dahlia	2.58	F	Mixed	Ball	E, 3
Dorado	1.92	M	Mixed	Plush toy	E, 1
Enzo B	4	M	Mixed	Plush toy	E, 3
Enzo K	1.92	M	Wheaten Terrier	Plush toy	C
Flossie	2.25	F	Mixed	Plush toy	C
Freja	7.08	F	Mixed	Plush toy	C
Gatsby	3.67	M	Havanese	Plush toy	C
Ginger	8.75	F	Mixed	Plush toy	E, 2
Gypsy	8.33	F	Mixed	Puzzle toy	E, 1
Jack	8.17	M	Mixed	Squeaky toy	E, 1
Jameson	9.5	M	Mixed	Plush toy	E, 2
Jasper	0.5	M	Affenpinscher	Socks	C
Joey	1.67	M	Mixed	Rope toy	E, 3
Koa	4.25	M	Mixed	Plush toy	E, 3
Kona	2.75	F	Mixed	Rope toy	C
Leo	5.25	M	Mixed	Squeaky toy	E, 3
Lola	6.92	F	Mixed	Plush toy	E, 2
Lolo	11.92	M	Shetland Sheepdog	Puzzle toy	E, 2
Lou	8.58	F	Border Collie	Squeaky toy	C
Lulu	4.67	F	Fox Terrier	Plush toy	E, 2
Molly	7.17	F	Mixed	Sandbox	C
Olive	2.17	F	Mixed	Puzzle toy	E, 3

Oliver	5.5	M	Mixed	Ball	E, 2
Penny	9.5	F	Mixed	Plush toy	C
Percy	1.58	M	Mixed	Squeaky toy	E, 1
Perry	4.58	M	Cavalier King Charles Spaniel	Ball	E, 1
Pixie	1.25	F	Mixed	Plush toy	C
Pogo	3.83	M	Mixed	Plush toy	E, 1
Ponyboy	8.75	M	Mixed	(none)	E, 2
Poppy	0.92	F	Mixed	Ball	E, 2
Posie	4.25	F	Shih tzu	Puzzle toy	E, 3
Sage-Galileo	2.08	F	Mixed	Puzzle toy	E, 3
Shadow	2.25	M	Newfoundland	Plush toy	C
Shea	8.58	M	Mixed	Squeaky toy	E, 1
Sprout	6.17	M	Brussels Griffon	Ball	E, 1
Suki	2.25	F	Pug	Squeaky toy	C
Willa	7.67	F	English Springer Spaniel	Plush toy	E, 1
Ziggy	3.83	M	Mixed	Plush toy	C
ZiShunka	2.42	F	Mixed	Plush toy	E, 1
Zora	2	F	Mixed	Ball	E, 3

Note: "E" = Experimental group; "C" = Control group
